# Sprayable solutions containing sticky rice oil droplets reduce western flower thrips damage and induce changes in *Chrysanthemum* leaf chemistry

**DOI:** 10.3389/fpls.2025.1509126

**Published:** 2025-01-28

**Authors:** Thijs V. Bierman, Hocelayne P. Fernandes, Young H. Choi, Sumin Seo, Klaas Vrieling, Mirka Macel, Bram Knegt, Thomas E. Kodger, Ralph van Zwieten, Peter G. L. Klinkhamer, T. Martijn Bezemer

**Affiliations:** ^1^ Above-Belowground Interactions, Institute of Biology Leiden, Leiden, Netherlands; ^2^ Natural Products Laboratory, Institute of Biology Leiden, Leiden, Netherlands; ^3^ Weerbare Planten, Aeres University of Applied Sciences, Almere, Netherlands; ^4^ Physical Chemistry and Soft Matter, Agrotechnology & Food Sciences Group, Wageningen University & Research, Wageningen, Netherlands

**Keywords:** thrips, *Chrysanthemum*, integrated pest management, rice oil, metabolomics, GC-MS, 1H NMR

## Abstract

Thrips are one of the most challenging pests in agricultural crops, including *Chrysanthemum*. In this study we tested via two plant assays whether solutions containing sticky rice germ oil (RGO) droplets could effectively trap thrips and lower thrips damage on *Chrysanthemum*. In the first assay, we additionally assessed the metabolomic effects of these RGO droplet sprays and thrips presence on plant chemistry via ^1^H NMR and headspace GC-MS on multiple timepoints to investigate which plant metabolites were affected by spraying and their potential relation to plant resistance against thrips. In the second assay, we tested the individual RGO solution constituents against thrips. Our results suggested that the adhesive RGO droplets were not effective as a physical trap as only three out of 600 adult thrips were caught at the achieved coverage. However, average thrips damage was still reduced up to 50% and no negative effects on plant growth were observed up to 25 days. Results from the second plant assay indicated that the individual constituents of the solution containing RGO droplets may have direct effects against thrips. Metabolomics analysis of sprayed leaves via headspace GC-MS and ^1^H NMR indicated that fatty acids and several volatile compounds such as 4(10)-thujene (sabinene), eucalyptol, *cis*-4-thujanol, and isocaryophyllene were highest on day 10, while sucrose, malic acid, *o*-Cymene, and 3-Methyl-2-butenoic acid were highest on day 25. Plants with thrips showed higher flavonoid, carbohydrate and glutamine acetic acid levels, and lower fatty acids and malic acid levels. RGO application increased the levels of fatty acids and alcohols present on top of and inside the *Chrysanthemum* leaves, while decreasing the concentrations of volatile compounds such as eucalyptol, chrysanthenone and eugenol in the *Chrysanthemum* leaves. Most interestingly, the thrips effect on the plant metabolome was no longer visible in RGO treated plants at the later harvesttime, suggesting that RGO application may overrule or prevent the metabolomic effects of thrips infestation. In conclusion, our study provides new information on how the application of a new plant-based plant protection product affects insect herbivores and alters crop phytochemistry for improved herbivore resistance.

## Introduction

*Frankliniella occidentalis* (Pergande), or western flower thrips, is a globally occurring pest of many horticultural crops and ornamentals (Reitz and Funderburk, 2012), including *Chrysanthemum* (*Chrysanthemum × morifolium*). Thrips cause damage directly by sucking the contents of plant cells, which can lead to stunted growth and overall reduced biomass, and cause damage indirectly via the transmission of plant viruses (De Jager et al., 1995). Due to their small size, thigmotactic behavior, rapid reproduction, and resistance to pesticides, thrips are a difficult pest to manage (Reitz, 2009). Billions of dollars’ worth of yield are lost annually to thrips and plant viruses in the USA alone (Nilon et al., 2021; Reitz et al., 2008). Although chemical pesticides have been vital to manage thrips and other pests (Reitz and Funderburk, 2012), their environmental impact and the increasing ability of pests to resist them has led to the consensus that pesticide use is unsustainable (Cloyd, 2016). As such, alternative pest control methods are urgently needed to expand the arsenal that growers use to control thrips and other pests.

Recently, we introduced a new way to utilize plant-derived oils for pest control: sprayable solutions containing adhesive droplets made from oxidized plant oils for trapping small arthropods (Bierman et al., 2024; van Zwieten et al., 2024). These sticky droplets can be made from different oils, including rice germ oil (RGO) which is often seen as a waste material. By spraying plants with sticky oil droplets, we may provide them with a mechanical defensive function, similar to how glandular trichomes that secrete sticky substances help to protect a large variety of vascular plants against herbivore attackers (LoPresti et al., 2015). So far, we only tested in Petri-dish assays if the sticky droplets could catch thrips and reduce their damage. In this study, we took the next logical step and investigated whether spraying *Chrysanthemum* plants with the sticky rice germ oil droplets could be an effective way to trap thrips and reduce thrips damage to full plants.

In addition to acting against pests directly, the application of plant-derived oils may affect the physiology of the plants that are sprayed. Plant-derived oils can cause phytotoxic responses, can affect plant growth, nutrient content, expression of plant compounds that are toxic to herbivores, and can induce the emission of herbivore repellent or predator attractive volatiles (De Almeida et al., 2010; Kesraoui et al., 2022; Verdeguer et al., 2020). The precise effects of plant-derived oils on plant physiology may depend on several factors, including the type of compound, its dose, the time after application, and the physiological age of the plant (Leiss et al., 2011; Razzaq et al., 2019).

Herbivores, including thrips, can also affect plant physiology, for example via the induction of chemical plant defense pathways such as the jasmonic acid and salicylic acid pathways (Escobar-Bravo et al., 2017). Metabolites induced by these pathways may be toxic or repellent to herbivores, or attract herbivore predators, which both may affect the feeding behavior and fitness of the herbivores (Dicke and Baldwin, 2010; Kessler and Baldwin, 2002; Walling, 2000). Combined with resistance screening, metabolomics approaches (advanced chemical profiling techniques) provide a useful tool to understand the relationship between plant chemistry and insect performance (Kim et al., 2011). For example, Mežaka et al. (2023) used GC-MS to investigate the phytotoxic effects of aqueous extracts of *Carum carvi* (caraway) seed distillation by-products and found that application of these compounds decreased certain green-leaf volatiles emitted by *Cucumis sativus* (cucumber) up to ten days after application. Using UHPLC-MS, Macel et al. (2019) found that monomeric and dimeric acyclic diterpene glycosides were linked to thrips resistance in *Capsicum* spp. (pepper). Leiss et al. (2009b) identified the phenylpropanoids chlorogenic acid and feruloylquinic acid as a thrips resistance factor in *Chrysanthemum* leaves via ^1^H NMR.

For many crops, it is still unknown which metabolites are related to increased herbivore resistance and in what way the application of pest control products and the presence of pests may interact to alter the concentrations of these metabolites over time. Therefore, in addition to assessing direct effects on thrips, we also investigated the effects of the application of our solutions containing sticky droplets on the plant metabolome, both in absence and presence of thrips at multiple timepoints. Two full-plant assays were performed with *Chrysanthemum* where plants were sprayed with solutions containing adhesive droplets or other control solutions and infested with thrips or not. In the first assay, thrips damage and plant growth were measured after ten and twenty-five days. The metabolomes of RGO sprayed plants and of control plants (sprayed with water) were analyzed using ^1^H NMR and headspace GC-MS. In the second assay measurements were done after twenty-five days and all plants were infested with thrips. Our main research questions were: (1) Do solutions that contain adhesive droplets made from plant-based oils that are sprayed on plants catch thrips and reduce thrips damage? (2) Does spraying of solutions containing sticky oil droplets affect plant growth? (3) In what way do the solutions containing sticky droplets affect the metabolome of *Chrysanthemum* and (4) Are there interactions between RGO spraying, harvesttime, and thrips presence to shape the plant metabolome? Before new crop protection products are applied on a large scale, knowledge on their effects on target pests, plant growth, and plant chemistry are essential to understand their mode of action and infer whether these products are suitable for commercial use.

## Materials and methods

### RGO solution

A sprayable solution containing adhesive RGO droplets was produced at Wageningen University & Research as described by van Zwieten et al. (2024). The solution contained 95.75 wt% tap water, 1.25 wt% oxidized RGO (type: R360) droplets, 1 wt% F-108 (a surfactant), and 2 wt% alginate. This RGO solution and a water control were tested in plant assay 1. The RGO solution and four control solutions: a 1% F-108 solution, 2% alginate solution, and a solution containing both 1% F-108 and 2% alginate, and a water control, were tested in plant assay 2.

### Insects

A colony of *Frankliniella occidentalis* (obtained from a greenhouse in the Netherlands) was maintained in plastic cages (60×60×40 cm) at Leiden University for over 15 years on *Chrysanthemum* cut flowers (cv. Baltica Yellow) as described by Bierman et al. (2024). Climate room conditions were 25°C, 60% RH (70% inside cages) and 16-8 light-dark photoperiod (fluorescent TL-light).

### Plants and growth conditions

Ten-day old *Chrysanthemum × morifolium* (cv. Baltica White) cuttings, rooted in small peat blocks (source: Deliflor B.V., Maasdijk, The Netherlands) were transplanted to 2.2 liter plastic pots filled with a mixture of 3:1 (v/v) autoclaved potting soil to vermiculite, with 2.5 g/l osmocote fertilizer pellets. Plants were grown in a climate room at 25°C day, 23°C night temperature, 70% RH and a 8-16 light-dark photoperiod (6:00am-10:00pm, fluorescent TL-light, 15,340 lm m^-2^). When plants were 25 days old, plant assays were performed at Leiden University under the same climate conditions as during the plant growth phase.

### Plant assay 1

Eighty *Chrysanthemum* plants of similar size (25 days old, 10 unfolded leaves, 14.3 cm [SD: 0.99] shoot length) were selected and divided into eight groups of ten plants. 40 plants were sprayed with 10 ml water per plant and 40 plants were sprayed with 10 ml solution containing RGO droplets using a paint spray gun (Einhell, Art. No. 41.330.00). Spraying was done from 20 cm above each plant under 1.4 bar airflow pressure. The spray pattern control valve was opened a quarter rotation. The flow control valve was opened two full rotations. Plant coverage was estimated from pictures of several leaves of five randomly chosen plants using ImageJ-Fiji V2.9.0 by counting adhesive droplets and by estimating the percentage leaf area covered (**Supplementary Figure S1**). After 30 minutes of drying, plants were placed on felt circles located on plastic dishes (25cm diameter) and covered with thrips-proof cages made of transparent plastic cylinders (50 cm height, 22.5 cm diameter) that were closed off at the top with lids made of plastic rings with 120 μm thrips-proof nylon mesh (Leiss et al., 2009a). Felt circles were kept moist during the experiment to water the plants and to prevent thrips from escaping. Per spray treatment, 20 plants were infested with 20 female adult *F. occidentalis* at the base of each plant. One group of ten plants of each combination of spray treatment and thrips was harvested after 10 days, the other ten plants after 25 days. The cages with plants were placed in the climate room in ten blocks containing one plant of each treatment and harvesttime (**Supplementary Figures S2A, B**).

At harvest, the number of thrips caught in adhesive droplets, total thrips damage and necrosis damage (brown spots) on all leaves were scored in mm^2^ and the shoot length (cm) and number of leaves were recorded. Then, for each plant a stack of sprayed leaves, leaf number 2 to 9 (counted from the bottom and up) was sampled in aluminum foil, flash frozen between 7 to 9 pm and stored at -80°C until freeze drying. After freeze drying, samples were ground into a fine powder using 2 ml Eppendorf tubes with 5 tiny iron balls and a Tissuelizer II Bead Mill (Qiagen, Hilden, Germany). The leaf powder would be used for ^1^H NMR and headspace (HS)- GC-MS analysis. The remainder of the shoot of each plant was oven-dried at 60°C to obtain the dry weight (g).

### Plant assay 2

Five groups of ten plants (25 days old, 11 [SD: 0.83] unfolded leaves, 15.5 cm [SD: 1.57] shoot length) were sprayed with 10 ml of one of five treatments: (1) water, (2) 1% F-108 solution, (3) 2% alginate solution, (4) 1% F-108 + 2% alginate solution, or (5) the full solution containing adhesive RGO droplets. Coverage (no. droplets and % leaf area covered) was estimated as before. Each plant was placed in a thrips cage, infested with 20 female adult thrips, and placed in the climate room in ten blocks with one plant per treatment. After 25 days, the number of thrips caught in adhesive droplets, total thrips damage (both on the adaxial and abaxial leaf side) (**Supplementary Figures S3A, B**) and necrosis damage (adaxial and abaxial), shoot length, number of leaves, and shoot total dry weight were recorded.

### Analysis of plant growth and thrips performance

RStudio (v. 4.3.1) was used for data analysis. *p* values < 0.05 were considered significant. Assumptions for normality and homogeneity of variance were tested using Shapiro-Wilk tests and Levene’s tests. For plant assay 1: Total thrips damage was ln-transformed to meet normality assumptions, and a two-way ANOVA was used with spraying treatment and harvesttime as factors. Shoot length and dry weight were analyzed using three-way ANOVAs with spraying treatment, thrips presence (thrips or no thrips) and harvesttime as factors. Significant interactions of two-way and three-way ANOVAs were followed by simple effects analysis via pairwise comparison of marginal means, corrected for family wise error using Bonferroni adjustment. For plant assay 2, total thrips damage, the proportion of total adaxial leaf damage to total leaf damage per plant, shoot length, and shoot dry weight were analyzed using one-way ANOVAs, followed by pairwise comparison using Tukey tests.

### ^1^H NMR analysis

The samples were prepared for ^1^H NMR analysis following the protocol described by Kim et al. (2010) with some modifications as follows. Per sample (five replicates), thirty mg of freeze-dried and ground leaf material was extracted by 15 minutes of ultrasonication in 1 ml of CD_3_OD-KH_2_PO_4_ buffer in D_2_O (pH 6.0, 1:1, v/v) containing 0.29 mM TMSP-*d_4_*) as an internal standard. The resulting extracts were centrifuged at 13,000 rpm and 300 µl of the supernatant was transferred to 3 mm NMR tubes. ^1^H NMR measurements were performed on a Bruker Avance-III 600 MHz standard bore liquid-state NMR spectrometer with operating frequency of ^1^H resonating at 600.13 MHz. A cryoprobe of type TCI H&F/C/N-D with Z gradient was used. The temperature was kept constant at 298 K. For internal locking, CD_3_OD was used. For each proton measurement, a 30-degree pulse of 2.64 msec at 5.5 W power with a fid resolution of 0.36 Hz, 64 scans with a relaxation delay of 1.5 secs, and acquisition time of 2.7 sec, in total taking 5 min to complete the measurement. The water signal was suppressed using a pre-saturation method and low-power selective irradiation at 0.3 Hz H_2_O at 4.87 ppm. Time domain data was transformed to the frequency domain by Fourier transformation with a window function of exponential function and a line broadening parameter set to 0.3 Hz for smoothening. The generated spectrums were manually phased, baseline corrected and calibrated to TMSP-*d_4_* at 0.00 ppm or HMDSO at 0.06 ppm using TOPSPIN V. 3.2 software (Bruker). The NMR spectra were bucketed using AMIX 3.9.12 (Bruker Biospin GmbH Rheinstetten, Germany). The bucketed data was obtained by integration of the spectra at 0.04 ppm intervals. The peak intensity of individual peaks was scaled to the total intensity recorded from δ 0.30 to δ 10.02. Due to the residual signals of HMDSO and CH_3_OH-*d_4_*, the regions δ 4.7 – δ 5.0 and δ 3.28 – δ 3.34 were excluded from the analysis.

### Headspace GC-MS analysis

Headspace-GC-MS measurements (five replicates) were performed using a 7890A gas chromatograph equipped with a 7693 automatic sampler and a 5975C single-quadrupole mass detector (Agilent, Folsom, CA, USA). Volatile compounds were separated on a DB-5 column: 30 m × 0.25 mm, 0.25 μm film (J&W Scientific, Folsom, CS, USA), using helium (99.9% purity) as a carrier gas at a flow rate of 1.6 ml/min. Freeze-dried, ground and homogenized leaf material (100 mg per sample) was placed into 20 ml glass headspace vials. Each vial was incubated at 100°C for 30 minutes, then 500 µl headspace was sampled and injected (split mode 5:1, 8 ml/min flow) into the gas chromatograph. The oven temperature was programmed starting at 70°C, held for 1 minute, then increased at 3°C/min to 100°C, re-increased to 220°C at 7°C/min, and finally increased at 14°C/min to 300°C, held for 3 min. The ionization energy in EI mode was 70 eV, and the mass scan range was set to 50-550 m/z. Tridecane (500 ng/ml) was used as an internal standard. The obtained HS-GC-MS data files of the samples were converted to.mzml format using the MSConvert tool from the Proteowizard software suite. The auto-deconvolution of GC-MS data and multivariate analysis were executed according to the workflow outlined by the Global Natural Product Social Molecular Networking (GNPS) platform (Global Natural Product Social Molecular Networking, 2024). The detected peaks were identified by comparison of their retention times and ion spectra with those listed in Atomic Spectra Database, NIST 14 (libscore cutoff value of 70). The data was then processed using MassHunter (B.07, Agilent). The obtained GC-MS data were processed by GNPS.

### Multivariate data analysis of HS-GC-MS and ^1^H NMR data

Principal component analysis (PCA) and orthogonal projection to latent structures discriminant analysis (OPLS-DA) were performed using SIMCA P (version 18.1, Sartorius). PCA was used to analyze the inherent variation within the datasets, with all data subjected to Pareto-scaling. OPLS-DA models were then used to assess variation in metabolite profiles between spray treatments and to discern distinct chemical compounds. The quality of the OPLS-DA models was estimated by R^2^X and Q^2^ values. Q^2^ values were obtained from permutation tests (100 permutations). R^2^X indicated the model’s fitness and was defined as the proportional variance, whereas Q^2^ was defined as the predictable variance (Villa-Ruano et al., 2019). S-plots were used to identify possible biomarkers.

## Results

### Effect of RGO application on thrips performance and plant growth over time

**Figure 1:**
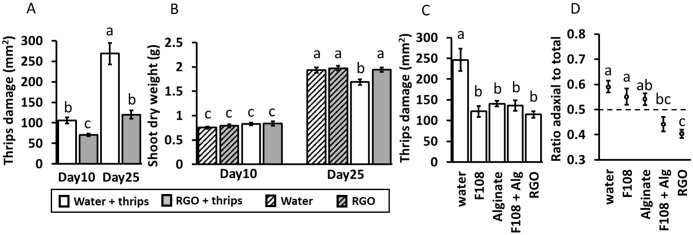
Plant assay 1 (n = 10): mean (± 1SE) total thrips damage (mm^2^) per plant **(A)**, and mean shoot dry weight (g) **(B)** of *Chrysanthemum* plants sprayed with water or solution containing adhesive RGO droplets. Plants were harvested after 10 or 25 days and were infested with thrips or not infested. Plant assay 2: mean (± 1SE) total thrips damage (mm^2^) **(C)**, and proportion of adaxial leaf damage to total leaf damage **(D)** after 25 days with thrips on leaves of *Chrysanthemum* plants sprayed with: water, 1% F-108 solution, 2% alginate solution, 1% F-108 + 2% alginate (F108 + Alg) solution, or an F-108 + alginate solution containing adhesive RGO droplets (RGO). Different letters indicate significant differences at *p* < 0.05 between treatment groups as found by two-way ANOVA (for **A**), three-way ANOVA (for **B**), both followed by comparison of marginal means or one-way ANOVAs followed by Tukey tests (for **C, D**).

Plant assay 1: RGO sprayed plants showed lower mean thrips damage than water sprayed plants, both after ten days (F_1, 36_ = 11.97, *p* < 0.01) and 25 days (F_1, 36_ = 49.12, *p* < 0.01; **Figure 1A**). Furthermore, thrips damage on water sprayed plants increased faster over time than on RGO sprayed plants (F_1, 36_ = 6.30, *p =* 0.02). However, only one adult thrips was observed to be stuck in an adhesive droplet. Coverage with adhesive droplets was also rather low with on average 159 (SD: 13) droplets per leaf (108 with area 0.1-0.5 mm^2^, 44 of 0.5-1 mm^2^, 7 of 1 mm^2^ or above) or 1.74% (SD: 0.1) of the total leaf area. The leaf surface also shimmered slightly and small white flakes were visible, indicating that some of the other compounds in the sprayable solution were also present on the leaf surface (**Supplementary Figures S4A, B**). Observation via a binocular confirmed droplets of presumably less oxidized rice germ oil were also present in the sprayable RGO solutions, likely being responsible for the shimmer (**Supplementary Figures S5A, B**).

As expected, shoot length (**Supplementary Figure S6**) was higher on day 25 than on day 10 (F_1, 72_ = 1601.64, *p* < 0.01). Spray treatment did not affect mean shoot length (F_1, 72_ = 2.26, *p* = 0.14). In general, plants with thrips had a lower shoot length (F_1, 72_ = 44.67, *p* < 0.01) and thrips presence reduced shoot length more on day 25 than on day 10 (thrips × day interaction: F_1, 72_ = 9.60, *p* < 0.01). Other two-way interactions and three-way interactions were not significant for shoot length. Dry weight (**Figure 1B**) was also higher for plants on day 25 than on day 10 (F_1, 72_ = 1266.26, *p* < 0.01). However, significant interactions were detected between thrips presence and harvesttime (F_1, 72_ = 10.74, *p* < 0.01) and between spray treatment and harvesttime (F_1, 72_ = 4.09, *p* = 0.047). Pairwise comparisons of marginal means indicated that, only for day 25, for plants infested with thrips, dry weight was less in water-treated plants compared to plants treated with solution containing RGO droplets (F_1, 72_ = 17.77, *p* < 0.01). For plants without thrips, no difference in treatments was visible on day 25 (F_1, 72_ = 0.42, *p =* 0.52) and the dry weight of these plants was similar to that of plants treated with RGO solution and infested with thrips.

Plant assay 2: Only two thrips were found stuck in adhesive droplets. Coverage with adhesive droplets was estimated at around 199 (SD: 15) droplets per leaf (141 with area 0.1-0.5 mm^2^, 46 of 0.5-1 mm^2^, 12 of 1 mm^2^ or above) or 3.3% (SD: 0.2) of the total leaf area. Thrips damage was higher in the water treatment than in all other treatments (F_4, 45_ = 12.23, *p <* 0.01; **Figure 1C**). The proportion of adaxial to total damage was highest in the water treatment, intermediate in the F-108, alginate and F-108 + alginate treatments, and lowest in the RGO treatment (F_4, 45_ = 9.95, *p* < 0.01; **Figure 1D**). No differences in mean shoot length (F_4, 45_ = 0.66, *p =* 0.62, **Supplementary Figure S7A**) or shoot dry weight (F_4, 45_ = 1.10, *p =* 0.37, **Supplementary Figure S7B**) were found between treatments.

### Effect of RGO droplets, harvesttime, and thrips on the metabolome of *Chrysanthemum* leaves as detected by ^1^H NMR

In this study, we used ¹H NMR to examine the metabolite profiles of *Chrysanthemum* leaves of RGO sprayed and control plants. As shown in **Supplementary Figure S8**, major detected compounds included flavonoids (such as apigenin glycosides, 5’,7’,3’,4’-tetrahydroxy flavanone glycosides, epi- or gallocatechin gallate), carbohydrates (glucose, fructose, stachyose, and sucrose), organic acids (including formic-, fumaric-, malic-, and acetic acid), amines (choline, betaine), amino acids (alanine, glutamine), triterpenoids, and steroids.

**Figure 2:**
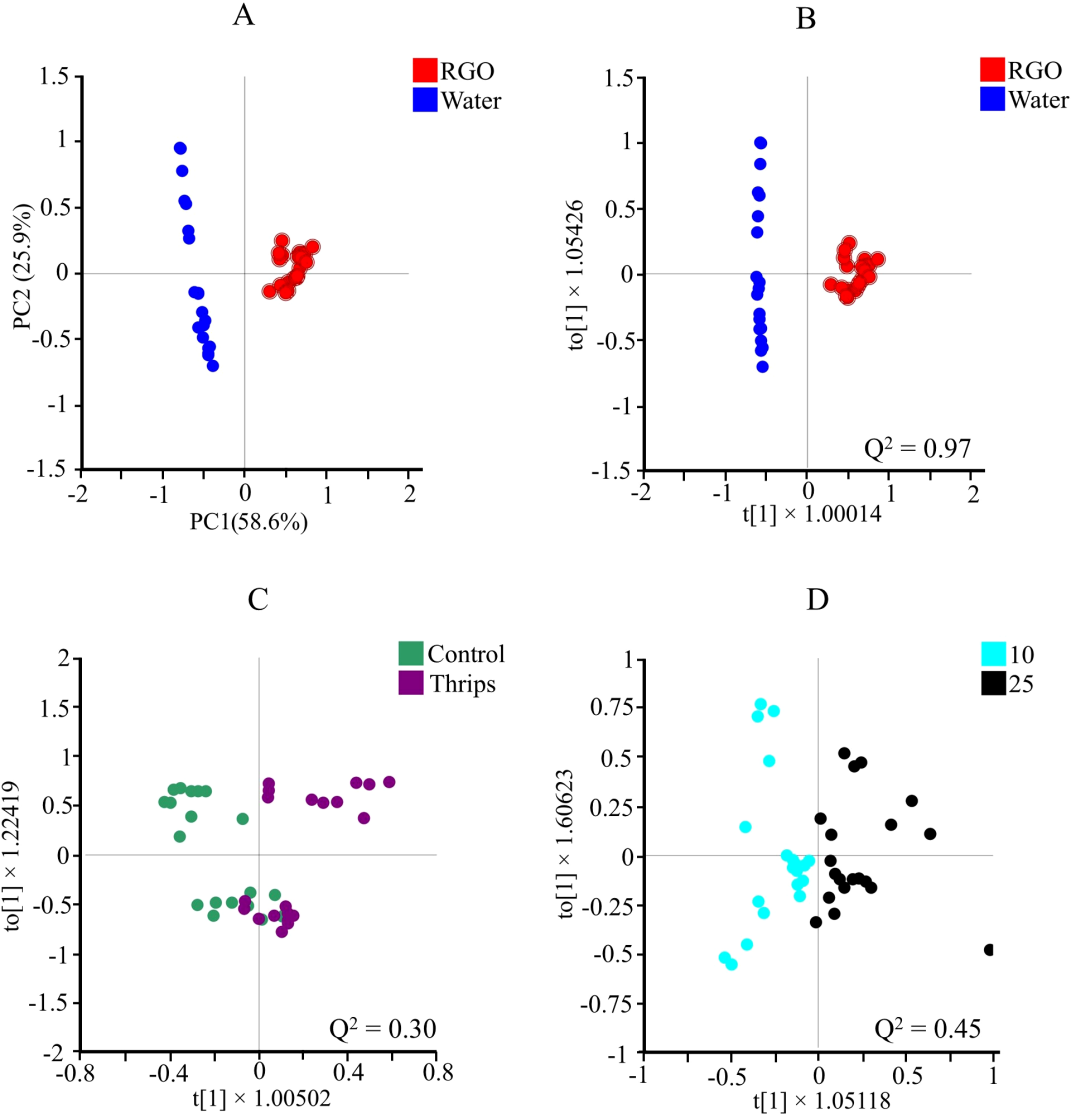
Score plots of principal component analysis (PCA, n = 10) **(A)**, and orthogonal partial least squares discriminant analysis (OPLS-DA) obtained from ^1^H NMR data using two classes of: RGO and water treated **(B)**, control and thrips treated **(C)**, and two different harvesting days after treatment (10 and 25 days after treatment) **(D)** of *Chrysanthemum* leaves. For each of OPLS-DA, Q^2^ values are depicted for each score plot.

After initial visual inspection of the ¹H NMR spectra, multivariate data analysis was conducted. The spectra were binned at intervals of 0.04 ppm, yielding 243 variables. Principal component analysis (PCA) was initially applied to the binned data to assess the primary factors influencing the *Chrysanthemum* leaf metabolome. As depicted in **Figure 2A**, the rice germ oil (RGO) treatment had the most significant impact on the metabolome, while other factors, such as harvesttime (10 vs. 25 days) and thrips infestation, were less prominent in the major principal components (PC1 and PC2).

**Figure 3:**
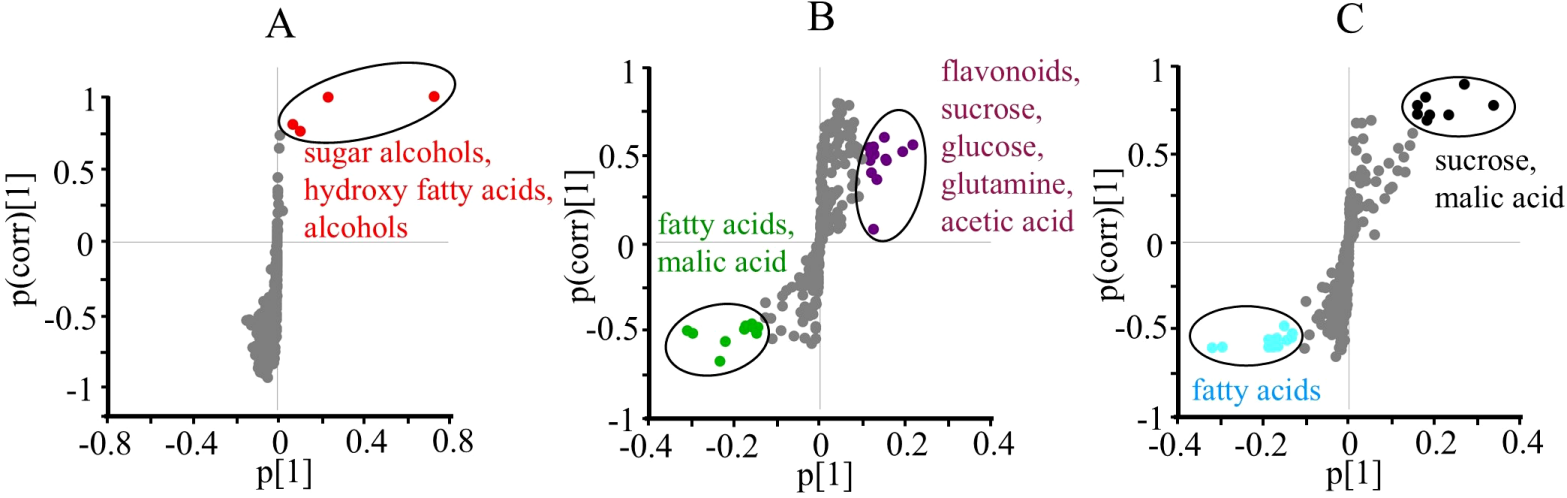
S-plots of orthogonal partial least squares discriminant analysis (OPLS-DA, n = 10) obtained from ^1^H NMR data using two classes of RGO and water treated **(A)**, control and thrips treated **(B)**, and two different harvesting days after treatment (10 and 25 days after treatment) **(C)** of *Chrysanthemum* leaves. Potential biomarkers are indicated with an oval outline and descriptive text.

To further investigate these minor factors, orthogonal partial least squares-discriminant analysis (OPLS-DA) was applied. The data were classified into three groups: (1) water vs. RGO treatment (**Figure 2B**), (2) control vs. thrips infestation (**Figure 2C**), and (3) harvesttime (10 vs. 25 days) (**Figure 2D**). The OPLS-DA results confirmed that the RGO treatment had a distinct impact on the metabolome, with a high Q² value of 0.97, while harvesttime also had a significant effect (Q² = 0.45). In contrast, thrips infestation showed only a marginal separation with a Q² value of 0.30 (**Figure 2C**).

To identify the specific metabolic changes associated with each factor, S-plots from the OPLS-DA models were examined (**Figure 3**). The RGO treatment was associated with higher levels of sugar alcohols assumed by the increase signal in the range of δ 3.5 – 3.7 and oxygenated fatty alcohols and acids likely present on top or inside of the leaves (**Figure 3A**). Thrips infestation led to increased levels of flavonoids, sucrose, glucose, glutamine, and acetic acid, but decreased levels of fatty acids and malic acid (**Figure 3B**). Regarding the harvesttime, older leaves (25 days) exhibited higher levels of sucrose and malic acid, while fatty acid levels were lower (**Figure 3C**).

Finally, to assess the potential interactions between RGO treatment and other factors, separate OPLS-DA models were constructed for water-treated and RGO-treated samples (**Supplementary Figure S9**). In RGO-treated leaves, the separation by harvesttime was more pronounced, while the effect of thrips infestation on the metabolome was reduced with a Q^2^ value of 0.14 (**Supplementary Figure S9**).

### Effect of RGO droplets, harvesttime, and thrips on the volatile headspace of *Chrysanthemum* leaves as detected by headspace GC-MS

**Figure 4:**
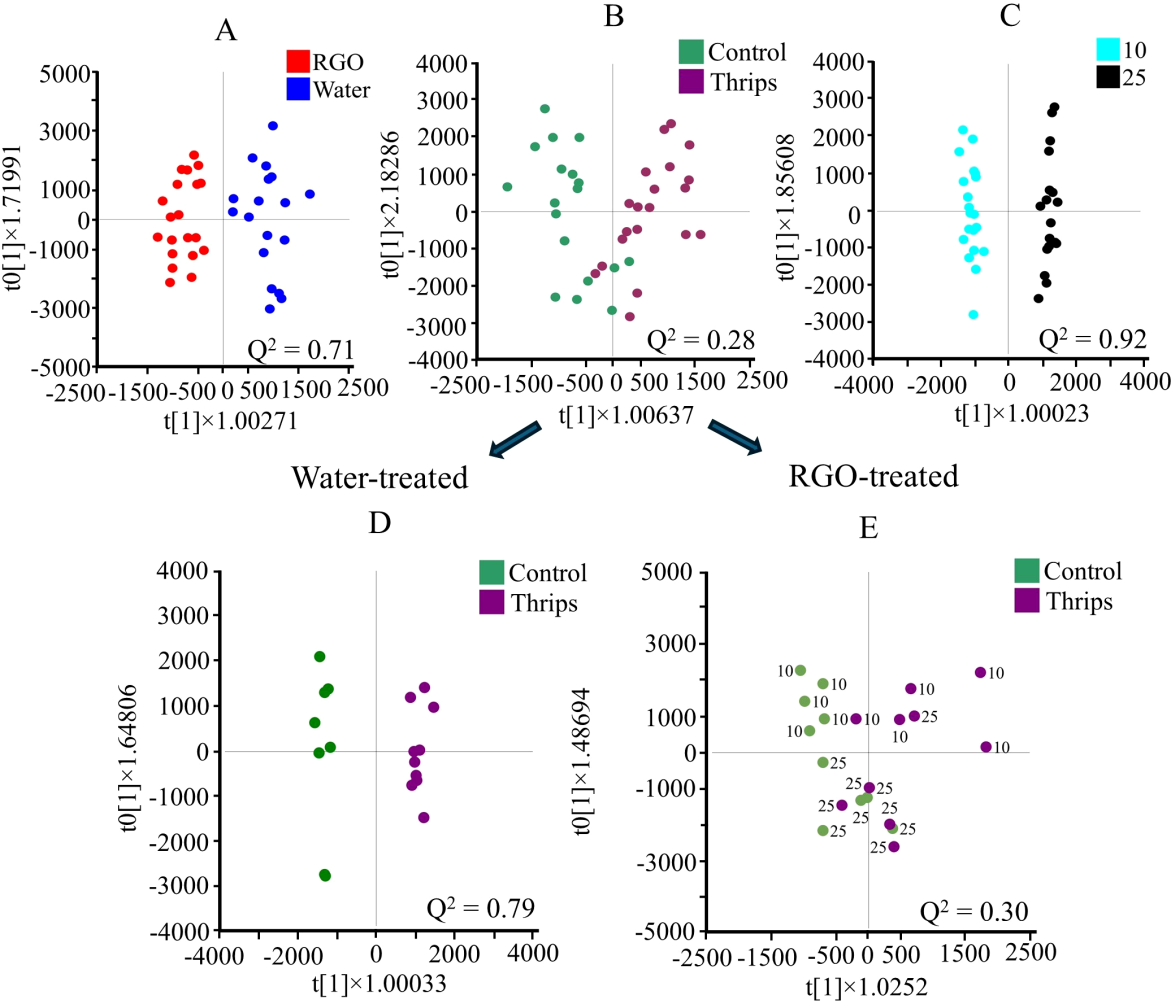
Orthogonal partial least squares discriminant analysis (OPLS-DA, n = 20) score plots obtained from headspace GC-MS data using two classes of RGO and water treated **(A)**, control and thrips treated **(B)**, and two different harvesting days after treatment (10 and 25 days after treatment) **(C)** of *Chrysanthemum* leaves. Additional OPLS-DA, n =10) analysis of the thrips factor for plants sprayed treated with water **(D)** and RGO **(E)**, separately. Numbers are used to indicate the harvesttime.

The use of ¹H NMR with CD_3_OD-KH^2^PO^4^ buffer extraction effectively identified a variety of metabolite groups influenced by different factors. However, some volatile compounds were not detected. To address this, headspace GC-MS was applied to the same set of *Chrysanthemum* leaves. A typical headspace GC-MS chromatogram is shown in **Supplementary Figure 10**. The resulting data, processed through GNPS for multivariate analysis, yielded 853 variables. The separation between groups in the GC-MS data (**Figures 4A–C**) was consistent with the results from ¹H NMR analysis. A notable difference, however, was that in the GC-MS analysis, the primary factor influencing the metabolome was harvesttime, whereas in ¹H NMR, RGO treatment was the dominant factor. Both methods revealed that thrips infestation had only a marginal impact, with a Q² value of less than 0.30 (**Figure 4B**).

Further analysis of the GC-MS data using S-plots of OPLS-DA allowed for the identification of characteristic metabolites associated with each factor. RGO treated leaf samples were generally found to have higher levels of octanal (most likely a component of the RGO) and lower levels of eucalyptol, chrysanthenone, and eugenol than those in water sprayed leaves. In terms of harvesttime, samples collected 10 days post-treatment exhibited higher levels of compounds such as 4(10)-thujene, *cis*-4-thujanol, eucalyptol, and isocaryophyllene, whereas samples collected after 25 days showed elevated levels of *o*-Cymene and 3-Methyl-2-butenoic acid.

Additional OPLS-DA analysis of GC-MS data, conducted separately for water- and RGO-treated samples, aimed to explore the relationship between RGO treatment, thrips infestation, and harvesttime (**Figures 4D, E**; **Supplementary Figure 11**). Similar to the ¹H NMR findings, RGO-treated *Chrysanthemum* leaves exhibited reduced differences between control and thrips-infested samples. The Q² value for control and thrips-infested RGO-treated samples was 0.30, compared to 0.79 in water-treated samples. The lower Q² value in the RGO-treated samples may indicate that the separation between control and thrips-infested samples diminishes over time, particularly after 25 days. This suggests that the RGO treatment becomes more effective later, around 25 days post-treatment (**Figure 4D**).

## Discussion

Natural materials hold a currently underutilized potential for use in crop protection. In previous work (Bierman et al., 2024), we showed that adhesive droplets made from oxidized rice germ oil (RGO) or other plant oils can catch western flower thrips and that the application of solutions containing these droplets may lead to lower damage and reproduction on detached *Chrysanthemum* leaves. In this study, we performed two full-plant assays to further investigate the effectiveness of the solutions containing RGO droplets to trap thrips and prevent thrips damage. Furthermore, we used ^1^H NMR and headspace GC-MS to study the effects of the RGO sprays on plant growth and the plant metabolome in presence and absence of thrips at different timepoints and tested the effects on thrips of the individual RGO constituents. The results of our study provide new insights into how plant-derived oils can be used directly for pest control, their effects on the metabolite content of plants, and how such metabolomic changes relate to levels of herbivory by thrips.

In nature, adhesive trichomes provide plants with a form of physical and chemical defense against herbivores and other insects (LoPresti et al., 2015). In line with our expectations, we were able to catch some thrips on plants covered with adhesive droplets, however, the number of caught thrips (three out of 600 adults that were released in RGO treatments) was very low, suggesting that the current RGO droplets are not effective for trapping thrips. Several factors in our experiment may explain these low thrips catch rates: the low density of 20 thrips per plant, a low coverage with (sufficiently sized) sticky droplets which were also only sprayed on the upper side of leaves, a potential decrease in adhesion of the RGO droplets over time, the ability of thrips to hide and feed on unsprayed and newly grown plant parts, and the potential ability of thrips to easily escape from the adhesive droplets once caught. Subsequent experiments with increased density of droplets on the plant, e.g., via increasing the concentration of droplets in the sprayable solutions or by performing multiple coatings with higher thrips densities and measurements at specific time intervals, are needed to determine to what extent a higher coverage of the droplets improves catch rate and for how long droplets remain effective. Since thrips may be repelled or attracted by plant-derived oils and their individual constituents (Koschier, 2008), experiments focusing on investigation of thrips behavior in the presence of the adhesive RGO droplets and their released volatiles may provide further insights into whether thrips actively avoid the droplets and to what extent volatile cues play a role in the trapping process.

Despite the low number of thrips caught, observed thrips damage was up to 50% lower and plant biomass loss was better prevented in plants sprayed with RGO solutions than in plants sprayed with water. The observed reduction in thrips performance suggests that, instead of the adhesive droplets acting as a physical trap, other mechanisms were likely involved, e.g., repellent or toxic effects of the droplets or other compounds in the sprayable solution, or chemical alterations in the plant or its surrounding headspace. Results from plant assay 2 support this hypothesis as the application of 1% F-108, 2% alginate, and F-108 + alginate solutions, and the full RGO sample were all found to reduce thrips feeding and cause a shift in feeding preference to the underside of the leaf. Results from prior leaf assays (Bierman et al., 2024) and earlier studies on effects of F-108 (Affeld et al., 2004), alginate (Saberi Riseh et al., 2022), rice germ oil (Rajput et al., 2017), and other plant-derived oils (Koschier, 2008) against arthropods also provide support that compounds in the spraying solution may have acted against thrips.

Upon stress, herbivore attack, or the application of natural compounds, plants commonly synthesize or release chemicals that may help to defend the plant (Kesraoui et al., 2022). The ^1^H NMR and HS-GC-MS analyses performed on the leaf material of plant assay 1 were a valuable approach to further investigate the effects of RGO application on the plant metabolome and headspace in relation to thrips performance over time. Many of the major ^1^H NMR and HS-GC-MS detected signals correspond to compounds such as flavonoids, carbohydrates, organic acids, amines, amino acids, triterpenoids, and steroids, which are commonly found in different *Chrysanthemum* cultivars and related species (Chae, 2016; Jiang et al., 2021; Kumar et al., 2005). The PCA and OPLS-DA plots showed clear effects on plant metabolism of RGO application, harvesttime, and to some extent thrips presence. RGO application increased hydroxy fatty acids and alcohols, while volatile compounds such as eucalyptol, chrysanthenone, and eugenol were decreased. Thrips presence was associated with increased flavonoid, carbohydrate, glutamine acetic acid levels, and lower fatty acids and malic acid levels. Sucrose, malic acid, *o*-Cymene, and 3-Methyl-2-butenoic acid, were highest after 25 days, while fatty acids and several volatile compounds such as 4(10)-thujene (sabinene), eucalyptol, *cis*-4-thujanol (an alcohol), and isocaryophyllene were highest after 10 days.

While the effect of harvesttime on metabolome content stayed consistent regardless of RGO application, more in depth analysis revealed that the thrips effect was more pronounced in water-treated plants than in RGO-treated plants. In the plants sprayed with RGO there was a considerable overlap in the multivariate plots at 25 days post infestation. A first explanation for this overlap may be that the RGO application induced a more general stress response that was unaffected by further stress from thrips as indicated by the increased levels of fatty acids, which are known to play a key role in general plant defense (He and Ding, 2020). A second explanation may be that the effect of thrips on RGO-treated plants diminished over time, perhaps as a result of decreased feeding which is known to be a factor in the severity of plant chemical responses to herbivores (Pan et al., 2021).

The altered concentrations of several of the compounds found on or inside the leaves of RGO-and water-sprayed plants and how the levels of these compounds changed over time and in presence of thrips, raises the question about their role in plant defense against arthropod pests. Flavonoids, such as kaempferol glucoside, are known to confer resistance to herbivory (Bennett and Wallsgrove, 1994; Chen et al., 2020; Leiss et al., 2009a). Carbohydrates have been reported to play a role in plant defenses as signaling molecules (Trouvelot et al., 2014) or as carbon resources for the sequestration of phenolic compounds (Arnold et al., 2004). Although some carbohydrates are used by herbivores for their own nutrition (Roeder and Behmer, 2014), an increase in general carbohydrate content may have deterred herbivory of thrips. Acetic acid has been shown to be involved in the jasmonic acid pathway and its application may induce plant herbivore and abiotic stress tolerance (Chen et al., 2019; Rahman et al., 2024). Fatty acids and malic acids have also been found in earlier work to be indicators of *F. occidentalis* resistance in tomato (Mirnezhad et al., 2010). Eucalyptol (1,8-cineole), a monoterpene, is a major constituent of *Chrysanthemum* (Chae, 2016) and is also found in eucalyptus tree leaves and other plant species like *Rosmarinus officinalis* (Koschier et al., 2002) or *Lavandula latifolia* (Erland et al., 2015). Eucalyptol has been found to have toxic activity against several arthropods such as the red flour beetle (Tripathi et al., 2001), house fly (Rice and Coats, 1994; Kumar et al., 2013), lesser grain borer (Ebadollahi et al., 2022), *Thrips palmi* (Kim et al., 2015) and *F. occidentalis* (Gharbi and Tay, 2022), although reports have also been made of 1,8-cineole acting as an attractant for *F. occidentalis* (Chermenskaya et al., 2001; Katerinopoulos et al., 2005; Koschier et al., 2000) and being negatively correlated with *F. occidentalis* mortality (Durr et al., 2022). Chrysanthenone, another major volatile constituent of *Chrysanthemum* (Chae, 2016), is suggested to have broader insecticidal activity (Kherroubi et al., 2021; Negahban et al., 2007). Eugenol, a fenylpropanoid, was found to attract *F. occidentalis* (Koschier et al., 2000), while also being an oviposition and feeding deterrent for *Thrips tabaci* (Riefler and Koschier, 2009) and deterrent of other insects such as mosquitoes (Nerio et al., 2010). α-Thujene (a monoterpene), of which 4(10)thujene (sabinene) and β-thujene are isomers, γ-terpinene, an isomer of β-terpinene, terpinolene, and δ-cadinene (a sesquiterpene) have all been found to be correlated with increased mortality of *F. occidentalis* (Durr et al., 2022). (E)-β-caryophyllene (an isomer of isocaryophyllene) is well known from studies in maize to be involved in resistance against some herbivore species, or as an attractant for other herbivores, nematodes, and for predatory insects (Degenhardt, 2009). The fact that we found decreased levels of eucalyptol, chrysanthenone, and eugenol in the leaves of RGO sprayed plants, and the fact that these compounds can be attractive to *F. occidentalis* seems to suggest that eucalyptol, chrysanthenone and eugenol are not the main drivers for conveying resistance to *F. occidentalis*. Instead, non-volatile constituents such as the presence of fatty acids on the leaves, carbohydrate content, malic acid, flavonoids and others may have contributed to the observed decrease in thrips damage. Further studies into the pathways and herbivore-antagonistic properties of the detected (volatile) compounds will be needed to gain deeper insight in their role in plant defense and to assess their potential for use in integrated pest management.

## Conclusion

New natural materials are being discovered and implemented in crop protection at a fast pace to replace synthetic pesticides. In this paper, we investigated whether sprayable solutions containing sticky droplets made from rice germ oil could be used for physical trapping of small arthropods such as thrips on plant surfaces and to what extent plant metabolomic changes induced by application of such oils may be related to increased crop resilience against thrips. While the solutions containing adhesive droplets did not act as an efficient trap, their application did reduce thrips damage substantially and caused altered levels of some metabolites such as flavonoids that are generally considered to be related to herbivore resistance. However, before spraying of solutions with sticky oil droplets is broadly implemented, further trials will first be needed to properly assess the droplet densities needed where spraying plants with solutions with adhesive droplets provides a reliable advantage, whether the droplets are compatible with natural enemies and pollinators, and to what extent their application affects photosynthesis, plant respiration and long-term growth, all of which may ultimately affect yield. The successful implementation of sprayable sticky plant oil droplets for arthropod trapping on crops will furthermore depend on the opinions of farmers and consumers about this method. Finally, NMR and GC-MS proved to be valuable tools in this study and will likely continue to prove their usefulness in the coming decades as a way to advance our knowledge of plant-insect interactions and to allow for this knowledge to be applied in agriculture e.g., via priming of plant defenses or breeding for arthropod resistance.

## Data Availability

The datasets presented in this study can be found in online repositories. The names of the repository/repositories and accession number(s) can be found below: https://zenodo.org/records/14604988.
