# Supplementary material for: Sprayable solutions containing sticky rice oil droplets reduce western flower thrips damage and induce changes in Chrysanthemum leaf chemistry
Source: Front Plant Sci. 2025 Jan 28;16:1509126. doi: 10.3389/fpls.2025.1509126 (PMC11811490; doi:10.3389/fpls.2025.1509126)
Supplement: Supplementary file 1 [file DataSheet1.pdf]

Supplementary Material

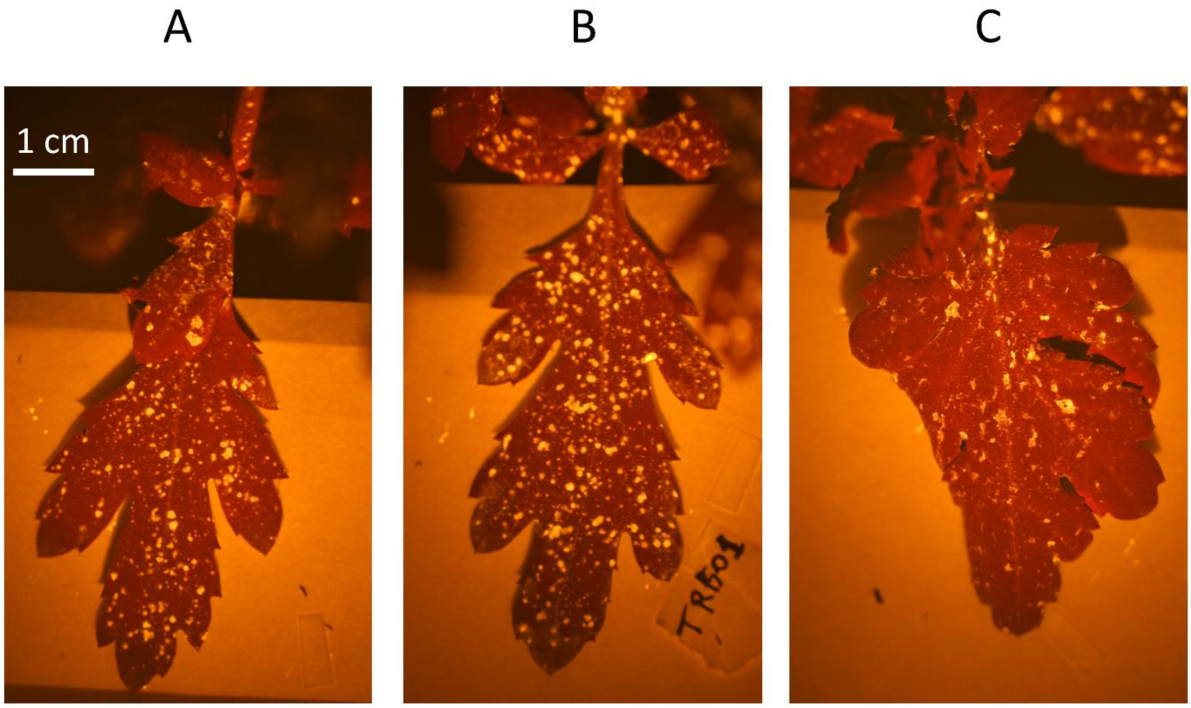

Supplementary Figure 1. (A B, C) *Chrysanthemum* leaves (left to right, leaf 2, 6, 10 as counted from the bottom of the shoot) covered with solutions containing adhesive droplets made from rice germ oil of plant assay 2, replicate 1. Pictures were taken under blue UV light (excitation between 440-460 nm) using a 570 nm LP filter.

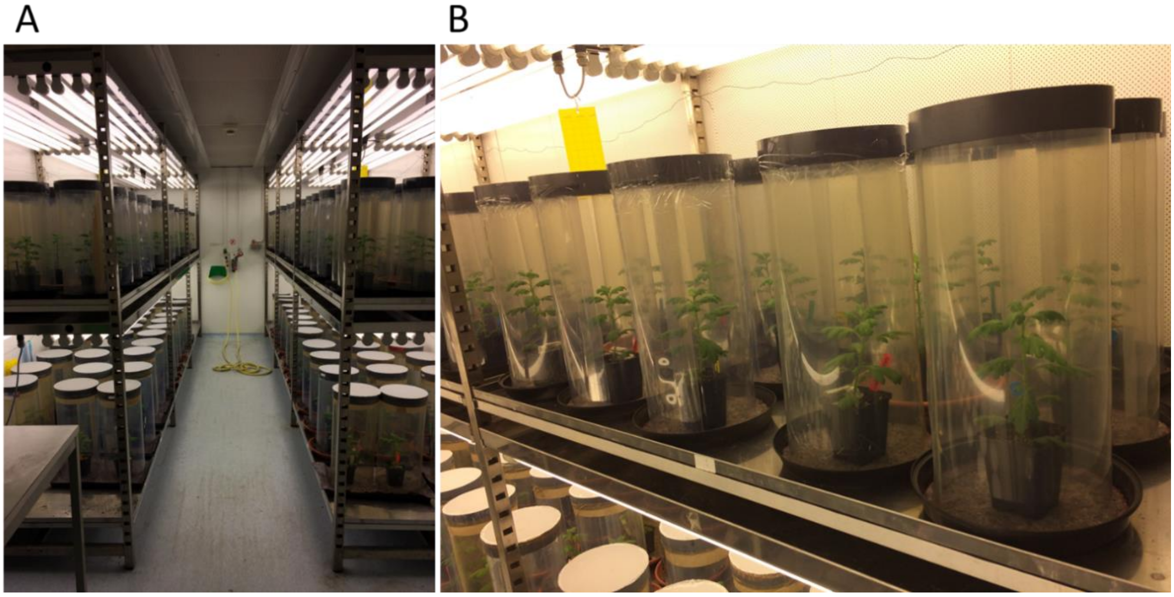

Supplementary Figure 2. (A, B) Climate room, experimental setup of plant assay 1.

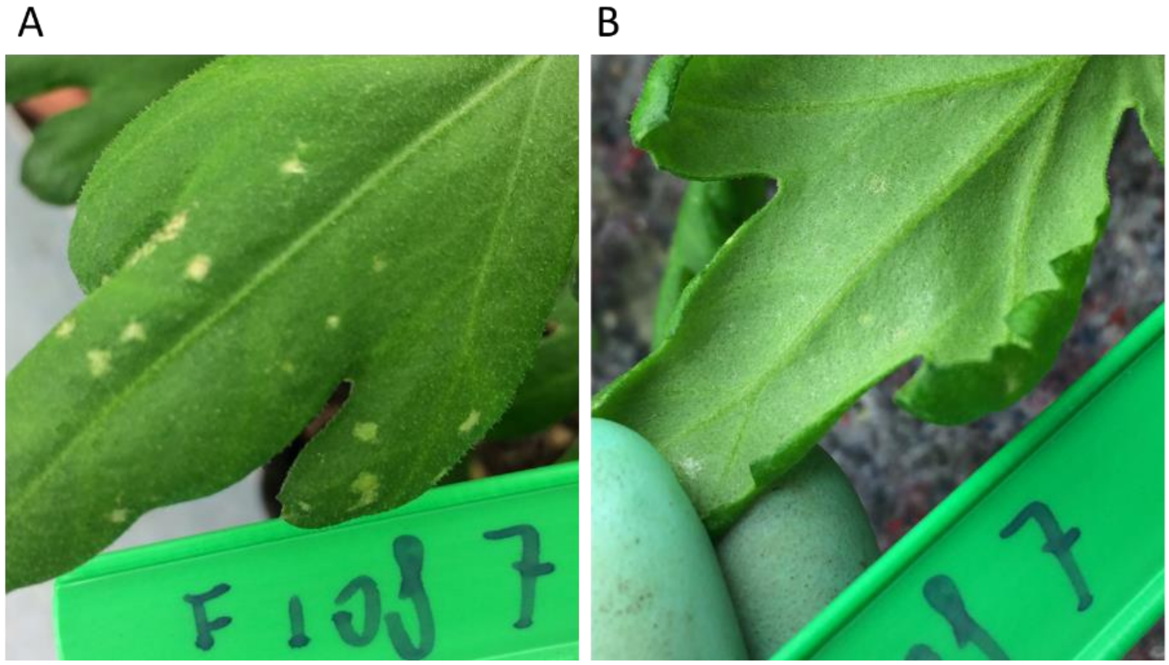

Supplementary Figure 3. Example of thrips damage on (A) adaxial leaf side, (B) abaxial leaf side.

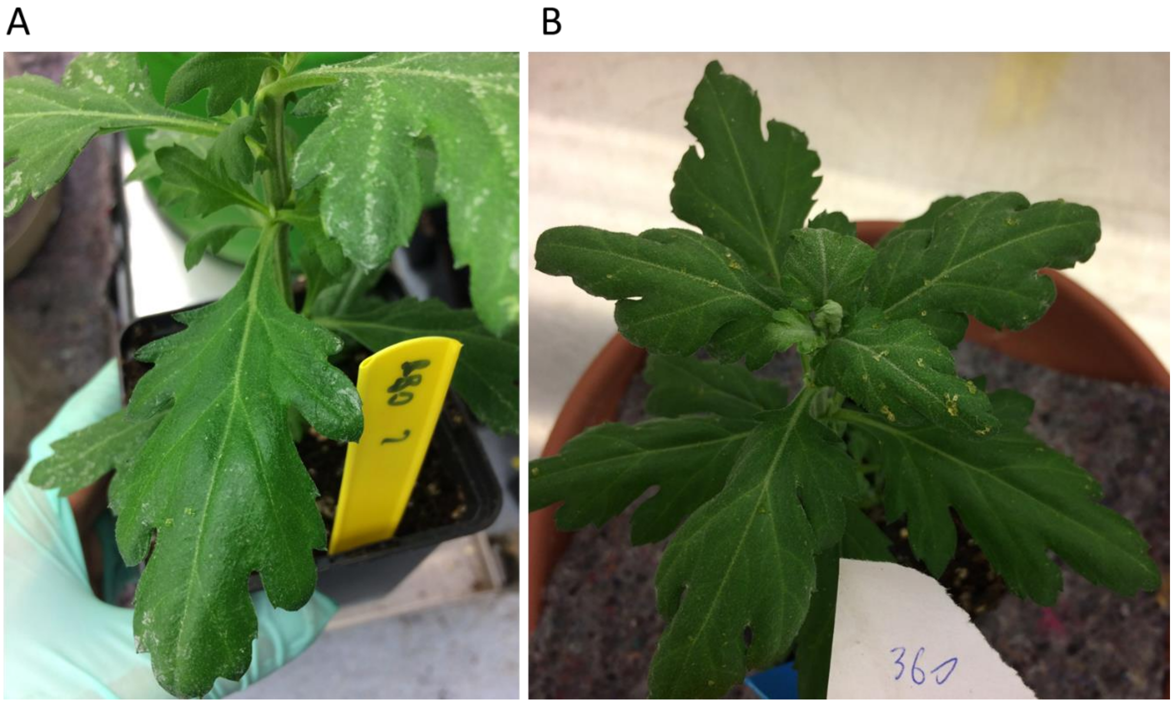

Supplementary Figure 4. Plant assay 1, (A) close up of chrysanthemum plant sprayed with solution containing RGO adhesive droplets. After drying, the alginic acid + F-108 can still partly be seen on the leaves as white areas, especially on younger leaves where leaf expansion seems to disrupt layers of dried up alginic acid + F108. (B) Coverage with droplets in this experiment was remarkably low, possibly due to droplets being washed off the leaves without prior drying steps as performed in later plant assays.

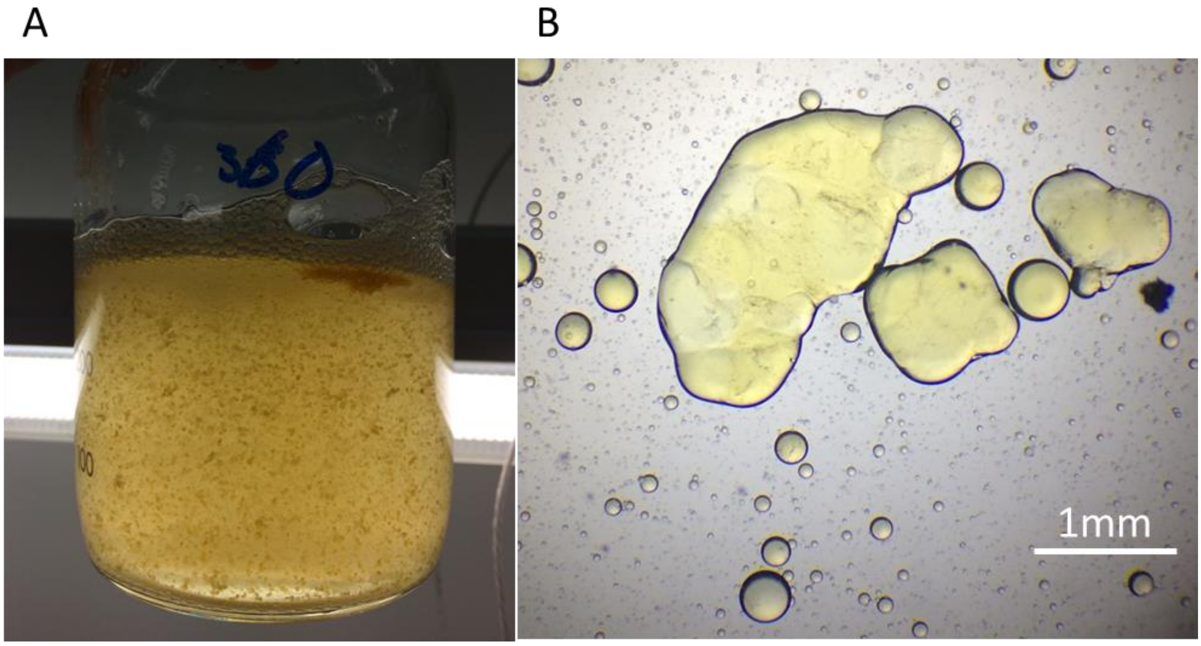

Supplementary Figure 5. (A) RGO (rice germ oil) adhesive oil droplets in alginic acid + F108 solution. (B) close up under binocular of RGO adhesive droplets revealing a more chunky appearance of the adhesive oil droplets. The circular droplets are likely non oxidized rice germ oil.

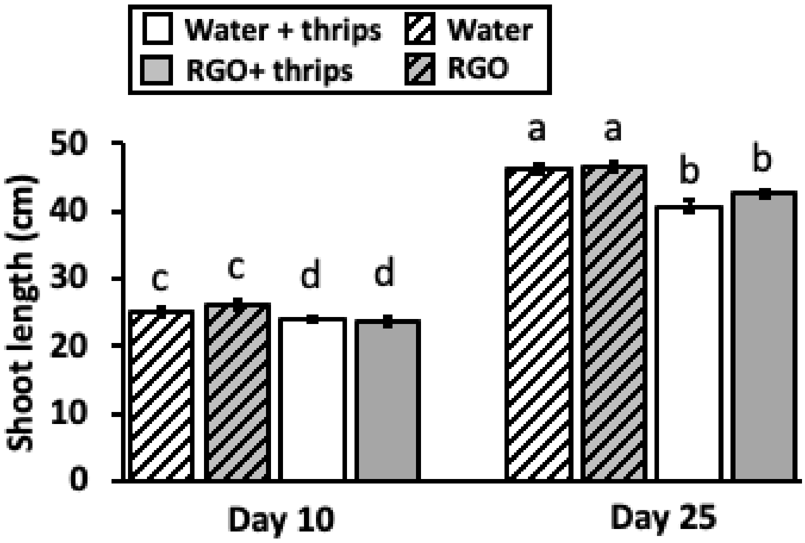

Supplementary Figure 6. Plant assay 1 (n = 10): mean (±1SE) shoot length of plants sprayed with water or solution with RGO adhesive droplets. Letters indicate significant differences between treatments as found by factor analysis using three-way ANOVA.

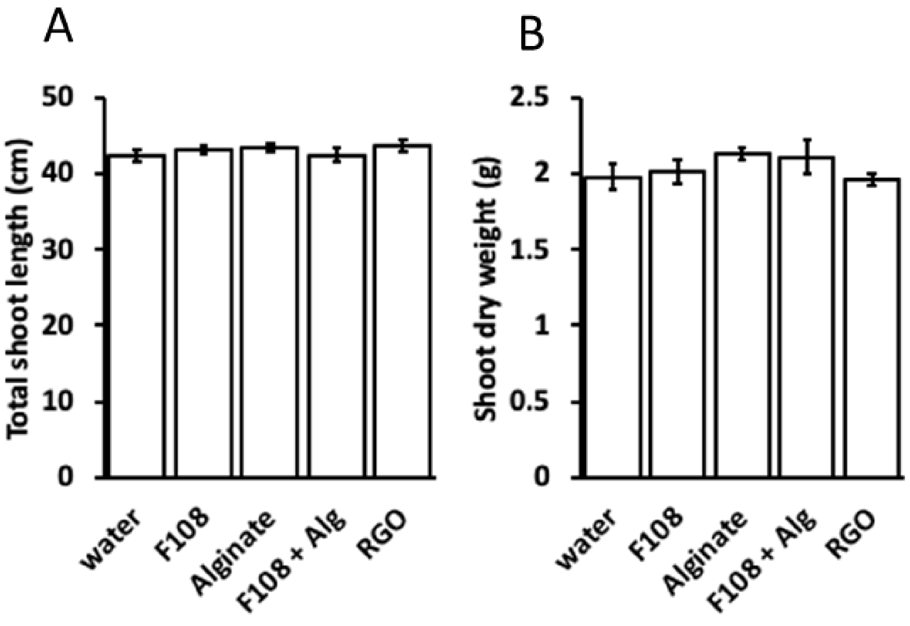

Supplementary Figure 7. Plant assay 2 (n = 10), mean (±1SE) shoot length (A) and dry weight (B) of plants sprayed with water, F-108, alginate, F-108 + alginate (solution), and solution with RGO droplets.

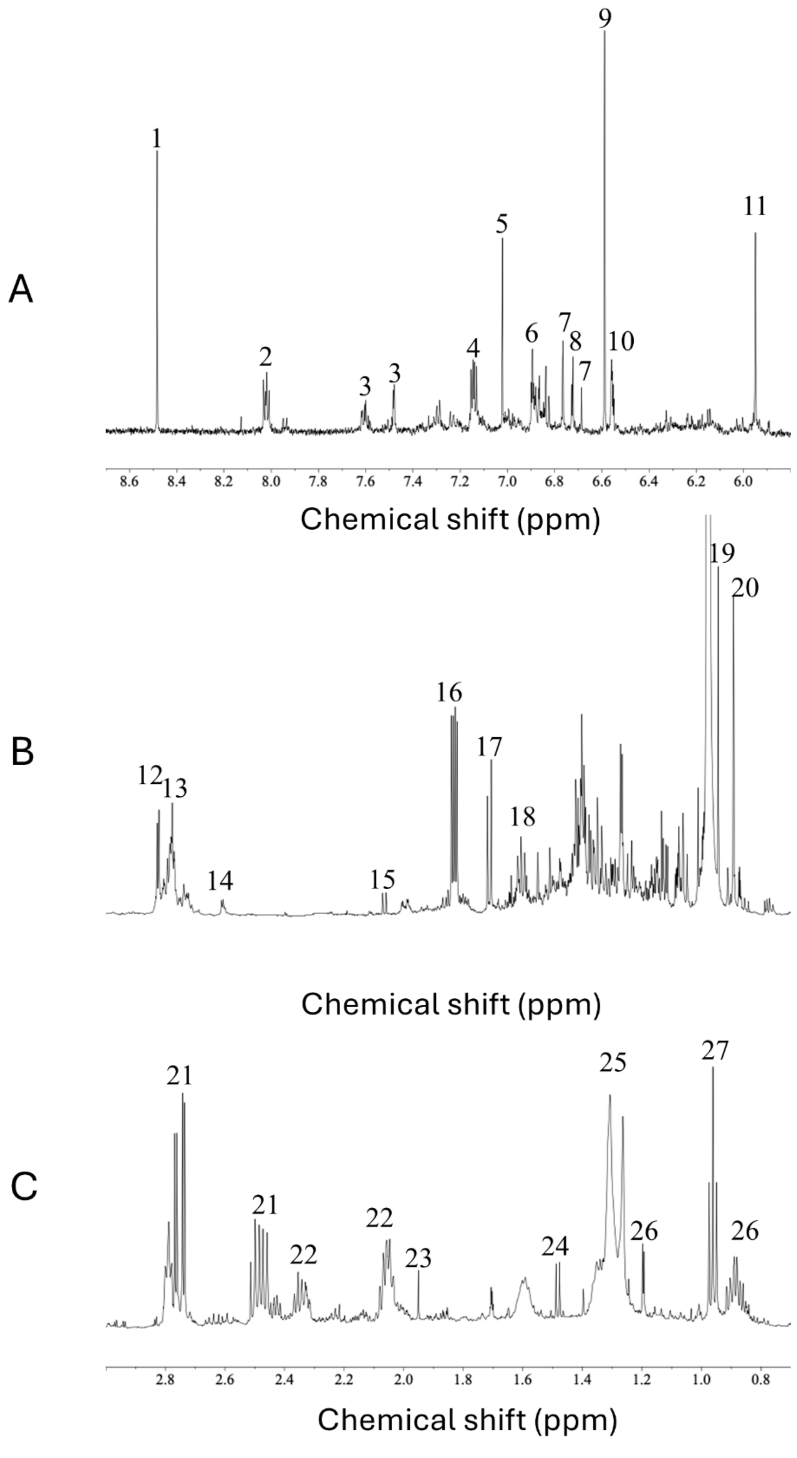

Supplementary Figure 8. Typical ^{1}H NMR spectrum of *Chrysanthemum* leaves in the range of δ 8.7 – 5.9 (A), δ 5.5 – 3.0 (B), and δ 3.0 – 0.7. 1: formic acid, 2: H-2’ and H-6’ of apigenin glycosides, 3: H-2’ and H-6’ of 5’,7’,3’,4’-tetrahydroxy flavanone glycosides, 4: H-3’ and H-5’ of apigenin glycosides, 5: gallate moiety of epicatechin gallate or catechin gallate, 6: H-5’ of 5’,7’,3’,4’-tetrahydroxy flavanone glycosides and H-3 of apigenin glycosides, 7: H-3’ of apigenin glycosides, 8, H-8’ of apigenin glycosides, 9: fumaric acid and H-2’ and H-6’ of epicatechin gallate or catechin gallate, 10: H-6’ of apigenin glycosides, 11: H-6’ and H-8’ of epicatechin gallate or catechin gallate, 12: H-1’ of sucrose, 13: H-1’ of stachyose and olefinic protons of terpenoids and fatty acids. 14: H-1’ of a-glucose, 15: H-1’ of b-glucose, 16: H-2’ of malic acid, 17: H-2’ of sucrose, 18: H-2’ of fructose, 19: betaine, 20: choline, 21: H-3’ of malic acid, 22: glutamine, 23: glutamine, 24: alanine, 25: -CH_2- of fatty acids, 26: CH_3 of terpenoids and steroids, 27: terminal CH_3 of fatty acids.

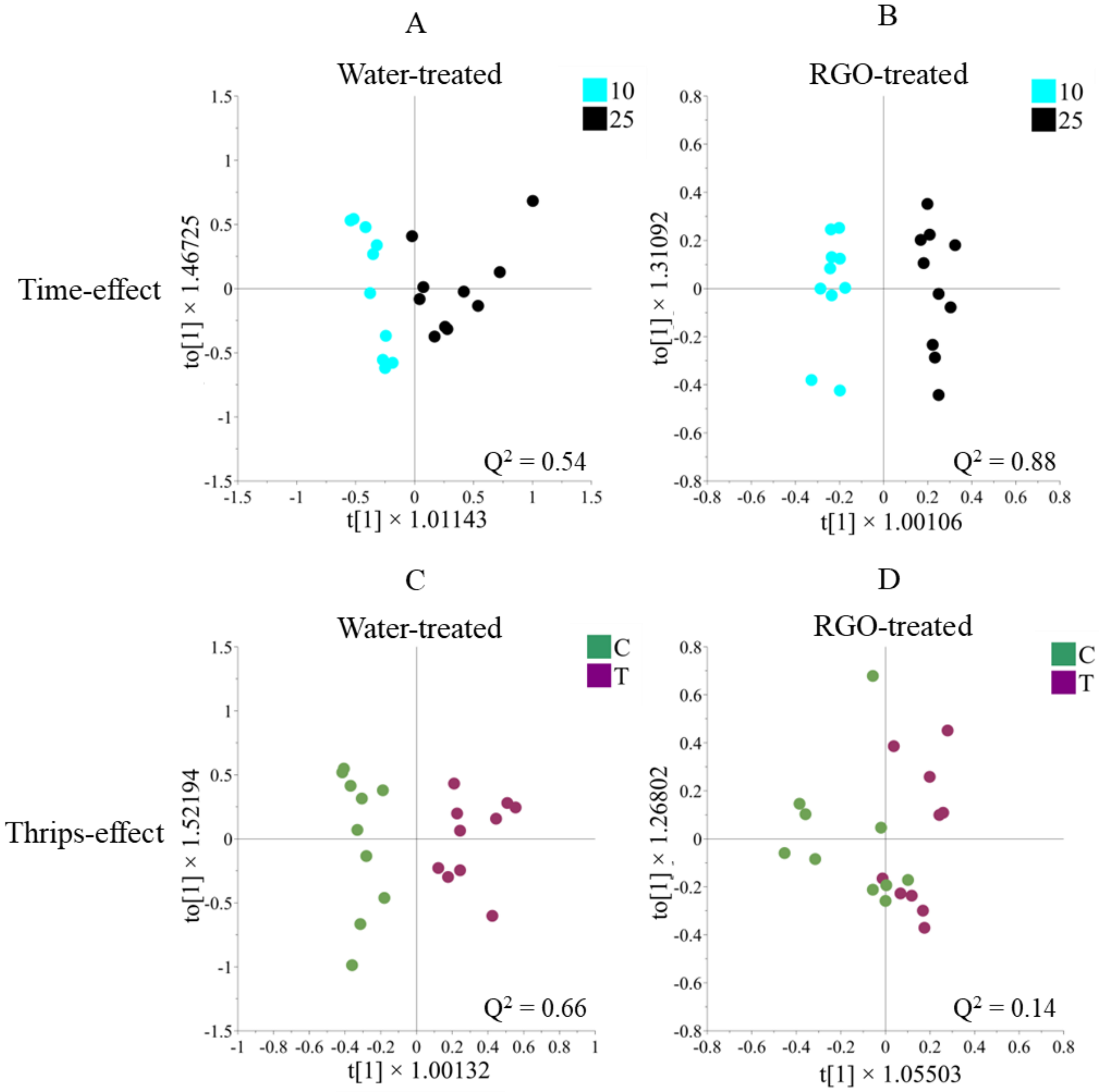

Supplementary Figure 9. Orthogonal partial least squares discriminant analysis (OPLS-DA, n = 10) score plots obtained from ^{1}H NMR data of water-treated (A) and RGO-treated (B) *Chrysanthemum* leaves separated by harvesttime of 10 and 25 days, and water-treated (C) and RGO-treated (D) control and thrips treatments.

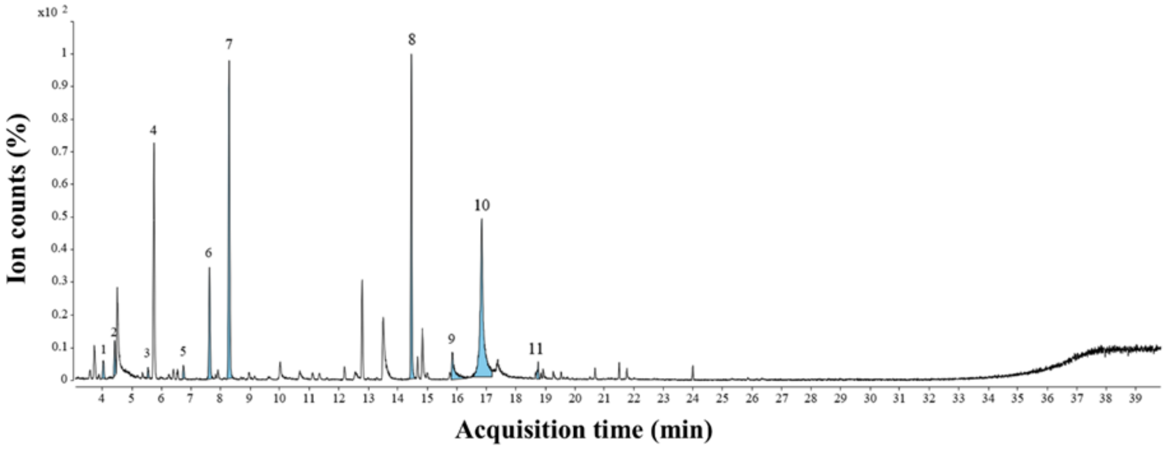

Supplementary Figure 10. Typical base peak headspace GC-MS chromatogram (BPC) of *Chrysanthemum* leaves. 1: 3-Methyl-2-butenoic acid, 2: 4(10)-thujene, 3: *o*-cymene, 4: eucalyptol, 5: *cis*-4-Thujanol, 6: chrysanthenone isomer, 7: chrysanthenone isomer, 8: tridecane, 9: eugenol, 10: chrysanthenone isomer, 11: isocaryophillene.

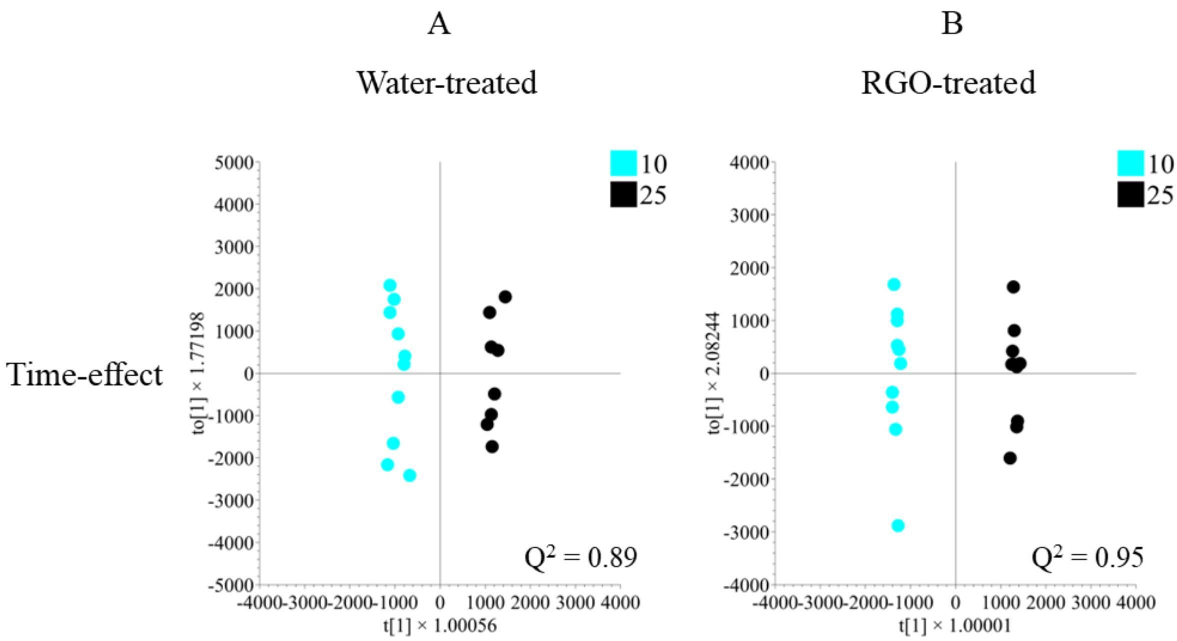

Supplementary Figure 11. Orthogonal partial least squares discriminant analysis (OPLS-DA, n = 10) score plots of the effect of harvesttime on the metabolome of water-treated (A) and RGO-treated (B) *Chrysanthemum* leaves obtained from headspace GC-MS data.
